# Solid-phase supported design of carriers for therapeutic nucleic acid delivery

**DOI:** 10.1042/BSR20160617

**Published:** 2017-10-31

**Authors:** Ana Krhac Levacic, Stephan Morys, Ernst Wagner

**Affiliations:** 1 Pharmaceutical Biotechnology, Center for System-Based Drug Research, and Center for NanoScience (CeNS), Ludwig-Maximilians-Universität München, Butenandtstrasse 5-13, D-81377 Munich, Germany; 2 Nanosystems Initiative Munich, Schellingstrasse 4, D-80799 Munich, Germany

**Keywords:** Artificial carriers, Nucleic acid delivery, Peptide-based carriers, Solid-phase assisted synthesis, Sequence-defined synthetic carriers

## Abstract

Nucleic acid molecules are important therapeutic agents in the field of antisense oligonucleotide, RNA interference, and gene therapies. Since nucleic acids are not able to cross cell membranes and enter efficiently into cells on their own, the development of efficient, safe, and precise delivery systems is the crucial challenge for development of nucleic acid therapeutics. For the delivery of nucleic acids to their intracellular site of action, either the cytosol or the nucleus, several extracellular and intracellular barriers have to be overcome. Multifunctional carriers may handle the different special requirements of each barrier. The complexity of such macromolecules however poses a new hurdle in medical translation, which is the chemical production in reproducible and well-defined form. Solid-phase assisted synthesis (SPS) presents a solution for this challenge. The current review provides an overview on the design and SPS of precise sequence-defined synthetic carriers for nucleic acid cargos.

## Introduction

Administration of nucleic acids with therapeutic potential offers a promising approach for the treatment of several human diseases that reached already for medical use [1–5]. Availability of efficient and safe delivery systems is of primary importance for wider spread of successful gene-based therapies. Due to large size, biodegradability and the negative charge of exogenous nucleic acids (NA) such as plasmid DNA (pDNA), mRNA, small interfering RNA (siRNA), microRNA (miRNA), or antisense oligonucleotides, transfer of therapeutic NAs to target cells requires help of viral and nonviral gene delivery systems. Although in current therapeutic clinical trials viral vectors dominate due to their higher efficiency, synthetic carriers show their advantages in the type of nucleic acid cargo (including also artificial chemically modified forms) [6,7], manner of production, formulation property, and storage [8–10]. Research on lipidic, peptide or polymer-based carriers that complex therapeutic nucleic acid by electrostatic interaction, is of particular interest for nonviral delivery. These vehicles should complex nucleic acids by formation of stabile polyplexes or lipoplexes [11] and protect against degradation in the bloodstream and reach target cells. The next requirement is an efficient intracellular delivery by entering via endocytosis into the intracellular space [9,12,13]. Endocytosis via invagination of nanoparticles by the lipid cell membrane into endosomal vesicles requests later escape from endosome instead of endolysosomal degradation [14–16]. In case of pDNA, either the whole polyplexes or the released nucleic acid must subsequently enter the nucleus via passive, active, or cell-cycle dependent mechanisms [17–21] and be transcribed [22]. Nucleic acids such as siRNA, miRNA, or mRNA need to reach the cytoplasm for bioactivity. Although on the one side, stability of complexes is important in the time of extracellular delivery steps, on the other side, the carrier should release the NA in the intracellular space and should not influence its functionality. Thus, for a successful nucleic acid delivery, synthetic nucleic acid shuttles have to be responsive to a changing bioenvironment just like natural viruses. Chemistry, size, and topology (linear, branched, comb, hyperbranched, and dendritic) of the shuttle, as well as size and physicochemical characteristics of formed nanostructures can play a decisive role for the biological activity [23–33]. For carrier optimization under such complex situations, a careful structure–activity relationship of carriers and their nucleic acid delivery characteristics is mandatory. This also requests synthetic methods to produce carriers in chemical precise form. One option outlined in this review presents the application of solid-phase assisted synthesis (SPS). Synthesis of peptides by SPS was introduced by Merrifield in 1963 [34] and has been refined to a very potent technology, which has been even applicable for the assembly of whole proteins such erythropoietin [35]. Analogous progress has been made in the area of SPS of oligonucleotides, applying phosphoramidite chemistry as initially developed by Caruthers [36]. Synthesis of oligonucleotides nowadays is routine; even the synthesis and subsequent recombinant assembly of oligonucleotides into a whole bacterial DNA genome was possible [37]. By nature of chemistry, nucleic acid analogs with favorable characteristics over their natural counterparts were generated [6,7,38]. In the current review, we focus on the design of nucleic acid delivery carriers that were synthesized by SPS. We summarize recent progress in the optimization of carrier design, differences between the most common groups of sequence-defined carriers, and their application for nucleic acid delivery and therapy.

## Solid-phase synthesis (SPS) of nucleic acid carriers

**Figure 1:**
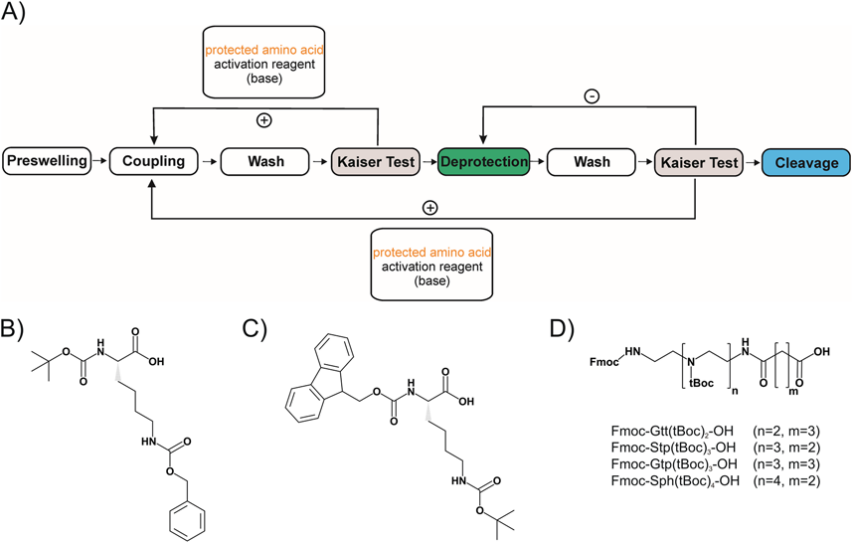
Precedure of SPS and exemplarily protected amino acids (**A**) Standard procedure of a solid-phase peptide synthesis cycle. tBoc as well as Fmoc strategy follow the same procedure of a repetitive coupling cycle. Resins are commonly swollen in DCM. Coupling requires activation of the carboxylic function of the amino acid either by carbodiimides or by formation of activated esters with PyBOP, HBTU, or HOBt and addition of DIPEA or TEA. Washing steps are performed with nonaqueous, peptide grade DMF, and DCM. A Kaiser test for detection of unprotected amines via ninhydrine reaction is done to verify successful coupling and deprotection. Nevertheless, tBoc and Fmoc strategies differ significantly regarding protecting groups, their removal (deprotection) as well as the final cleavage from the solid support. In tBoc strategy, α-amines of amino acids are tBoc protected, removal after coupling is performed with TFA and the final peptide cleavage is conducted with HF. In Fmoc strategy, α-amines of amino acids are Fmoc protected, removal after coupling is performed with a mixture of piperidine/DMF and the final peptide cleavage is conducted with a cleavage cocktail mainly consisting of TFA. (**B**) Protected lysine for tBoc (tBoc-L-Lys(Cbz)-OH) or (**C**) Fmoc (Fmoc-L-Lys(tBoc)-OH) strategy. (**D**) Fmoc, tBoc protected artificial oligoamino acids derived from polyethylenimine (PEI) repeating units; Cbz, benzyloxycarbonyl; Fmoc, 9-fluorenylmethoxycarbonyl; Gtp, glutaroyl-tetraethylene pentamine; Gtt, glutaroyl-triethylene tetramine; Sph, succinoyl pentaethylene hexamine; Stp, succinoyl tetraethylene pentamine; tBoc, tert-butyloxycarbonyl.

As outlined above, potent nucleic acid vehicles need to be bioresponsive and multifunctional. To enable clear-cut structure–activity relationships to be drawn, synthesis of precise carriers is needed. With improved chemistries, such as controlled radical polymer syntheses or specific ligation strategies, products with decreased polydispersity, and more controlled architectures of carriers can be achieved than before [39–44]. Nevertheless, the exact numbers of monomers and locations of attached subunits still are not under perfect control. As alternative, a series of researchers have applied the well-established method of SPS (see Figure 1) for developing linear [45–55] and branched [56–62] peptide-based, lipid-based [63–66], or artificial oligomer-based [66–78] sequence-defined nucleic acid carriers.

Merrifield introduced SPS as an alternative to liquid-phase peptide synthesis by selecting preactivated polystyrene as solid support for synthesis [34]. The first protected amino acid (AA) was loaded onto the resin. Orthogonally protected amino acids are coupled sequentially, with easy washing steps between coupling and removal of the protection group of the primary amine to constantly grow the macromolecule on the solid support, until the peptide and the remaining protecting groups (see Figure 1). In comparison with solution-phase synthesis, solid-phase synthesis offers three important advantages. First, easier purification of intermediates is possible, by simple removal of unreacted reagents by washing during synthesis. Second, reduction of side products (such as by repeated couplings or capping) leads to increased product yields. And third, due to the repetitive nature of the process, the whole assembly can be performed in automated form by peptide synthesizers.

The general procedure of a SPS cycle can be found in Figure 1A. Initially, tBoc chemistry was applied to the amino acid to protect the α-amino group. A solid support resin was introduced to sequentially assemble peptides [34]. The first amino acid with a tBoc α-amine (see Figure 1B) is linked to the solid support via the free, C-terminal carboxy group. The resin-bound amino acid is then treated with trifluoro acetic acid (TFA) to remove the tBoc protecting group and to free the α-amine. Now, the next tBoc protected α-amino acid can be coupled. For sequential amino acid coupling, the carboxylic acid group of the AA needs to be activated, most commonly by addition of dicyclohexylcarbodiimide (DCC). Dichloromethane (DCM) and dimethylformamide (DMF) are used as organic solvents, to create the required, nonaqueous environment for successful coupling, while facilitating swelling of the solid support during the reaction. At the end of the synthesis, the resin is treated with hydrofluoric acid (HF) to cleave the peptide from the resin and to remove all side chain protecting groups.

Replacement of classical tBoc chemistry by introducing the base-labile protecting group Fmoc (N-α-9-fluorenylmethyloxycarbonyl) [79–82] opened peptide manufacture to a wide range of operators as it no longer requires the application of the hazardous HF deprotection chemistry. The use of resins with novel acid labile linkers, like the hydroxymethyl-based Wang resin, the Rink amide resin, or the trityl chloride (especially 2-chlorotrityl chloride) [82] facilitated cleavage from the resin with TFA instead of HF. Instead of tBoc, Fmoc is now used to protect the α-amine of the amino acids (see an example in Figure 1C), which can easily be removed by non-nucleophilic bases like piperidine or diazabicycloundecene (DBU), while side chains of the amino acids are most commonly protected by acid labile protecting groups such as tBu, Trt, tBoc, or Pbf [83]. Also, sophisticated strategies for orthogonal synthesis of peptides are available [84]. The loading of the first Fmoc-protected AA onto the resin remains the initial step in Fmoc-peptide synthesis. After AA loading, free linkers on the resin need to be capped (e.g. with methanol) and the amount of AA loading needs to be determined by correlation of Fmoc absorbance [82]. The synthesis can then be continued in a defined matter by Fmoc deprotection of the first AA with a mixture of piperidine/DMF. The solvent, containing the cleaved Fmoc-group, is removed and the resin is washed with DMF and DCM. Then, the next Fmoc protected amino acid can be coupled. For this purpose, an excess of amino acid, and a weak, non-nucleophilic base like DIPEA (*N,N*-diisopropylethylamine) or TEA (triethylamine) are required. On the one hand, the base ensures a deprotonation of the amine. On the other hand, it deprotonates the carboxylic function to facilitate an easier active ester formation with an activation reagent. An activation reagent like PyBOP, HBTU, or HOBt, also preventing racemization, is usually dissolved in DMF to maintain nonaqueous environment. After coupling, the solvents are discarded, and the resin is washed with DMF and DCM. A ninhydrin reaction, detecting unreacted amines, can be now applied to validate successful amino acid coupling. This so-called Kaiser test [85] is based on the formation of a purple amino–ninhydrin complex (Ruhmanns purple), if free amines are present. After successful coupling, five cycles of Fmoc deprotection with piperidine/DMF are commonly applied. This cycle, from coupling to Fmoc removal, is conducted until the desired peptide is assembled. The resin is then dried and, dependent of the side chain protecting groups, a TFA-based cleavage cocktail, supplemented with the required scavengers like TIS (triisopropylsilane), water, or EDT (ethanedithiol), and the maintained products are purified via common purification methods such as SEC (size-exclusion chromatography).

Recently, the Fmoc peptide SPS strategy has been adopted for the synthesis of sequence-defined oligo(ethylenamino)amides (Figure 1D). Instead of natural amino acids, artificial oligoamino acids such as Stp (succinoyl tetraethylene pentamine), Gtp (glutaroyl-tetraethylene pentamine), or Sph (succinoyl pentaethylene hexamine) in Fmoc, tBoc-protected forms [75,86,87] can be used for manual or automated SPS, the latter requires a peptide synthesizer [88].

## Carrier requirements: nucleic acid binding

**Figure 2:**
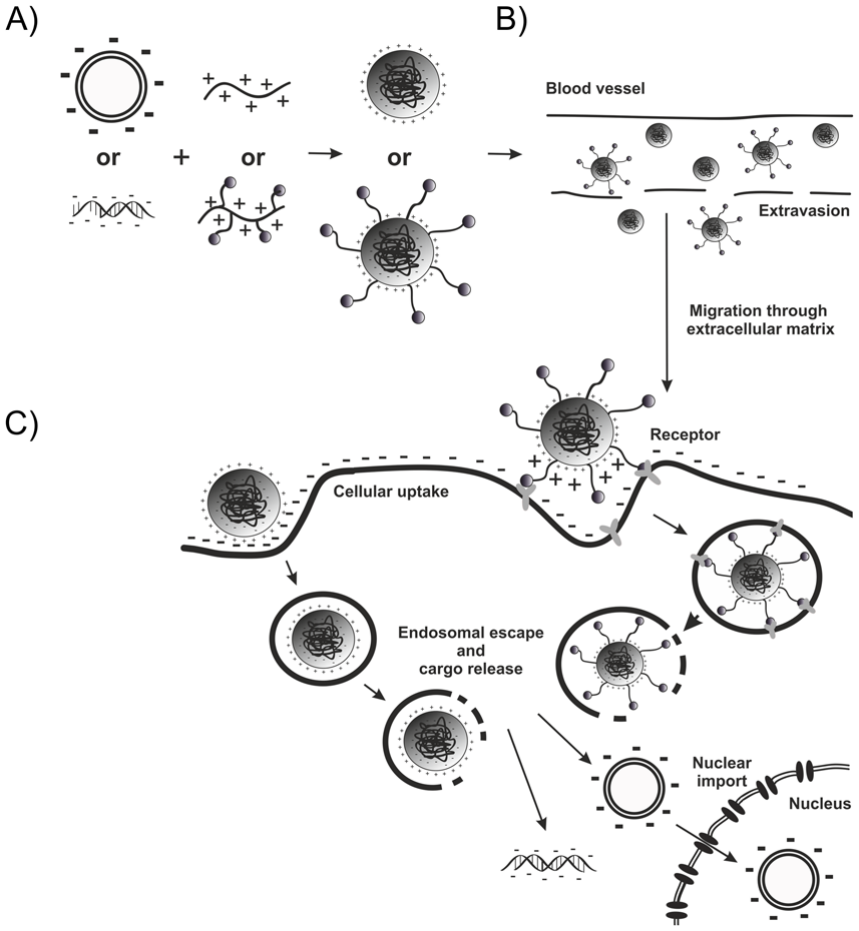
Barriers for the nucleic acid delivery via polyplexes (**A**) Formation of stable polyplexes. (**B**) Protection against rapid clearance and unspecific interactions with blood components, and (**C**) overcoming cellular barriers

**Figure 3:**
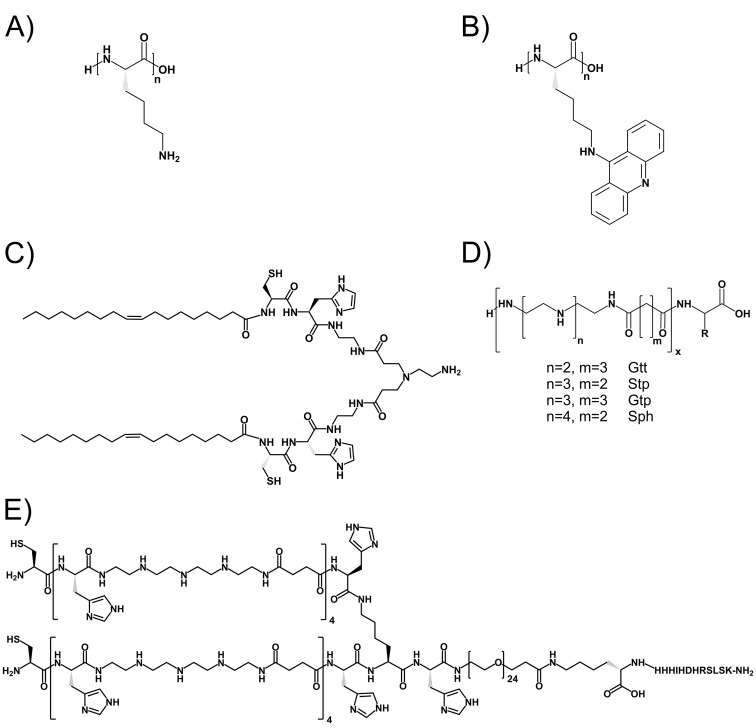
Oligopeptides and oligomers with nucleic acid binding motifs generated by SPS Nucleic acid binding motifs of (**A**) oligolysine and (**B**) acridine-modified oligolysine. (**C**) EHCO, a lipopeptide containing oleic acid, histidine, cysteine, and artificial aminoethyl blocks for nucleic acid binding. (**D**) Artificial amino acids derived from PEI repeat unit (see Figure 1) that are assembled by SPS to retrieve a nucleic acid binding domain within sequence-defined oligomers. (**E**) Example of HGFR/cMet targeted PEG-2-arm oligomer.

The multiple requirements for carriers to successfully deliver nucleic acids are described in Figure 2 in schematic form. Nonviral carriers tailored by solid-phase synthesis can be composed either solely of natural amino acids, solely of artificial building blocks, or of a combination of both (Figure 3). Especially homopolymers of the basic amino acid residues lysine (Figure 3A), ornithine, and arginine had shown ability to bind and condensate nucleic acid [89–92]. Later on, instead of polymerized amino acids, defined oligopeptides were developed via SPS [45–47,49,56]. Linear [45] as well as branched [56] oligolysine peptides were evaluated regarding nucleic acid binding and compaction as well as gene transfer. A minimum of six to eight cationic amino acids are required to compact pDNA into polyplexes active in gene delivery. The DNA binding and compaction ranked from arginine > lysine ∼ ornithine residues. Nucleic acid binding represents only one crucial step for successful gene delivery; not surprisingly, despite good nucleic acid binding oligolysine peptides could mediate gene transfer only to a limited extent, because of insufficient endosomal escape (see subsequent section). In several cases, combination with lysosomatropic chloroquine or lipidic helper molecules was necessary to mediate successful nucleic acid delivery [55,93–95].

Branched peptides containing α,ε-modified lysines as branching points, and lysines and protonatable histidine as nucleic acid binding arms were found as very effective in either pDNA or siRNA transfer [57–61]. It had been observed that the type of nucleic acid cargo strongly influences the carrier performance [55,96,97]. Interestingly, combinatorial work pointed out that little changes in topology can decide on whether the carriers is effective for pDNA or siRNA delivery [60,61]. These peptides with incorporated histidines had significantly decreased cytotoxicity as compared with classical transfection polymers [98].

Introduction of cysteines into oligolysine peptides offered a biodegradable and cross-linking motif that allowed polymerization of Cys-Lys_10_-Cys corresponding to polylysine Lys_205_ [50,51]. Analogously, increased pDNA binding was obtained by introduction of cysteines via SPS into Trp-Lys_18_ peptides, which led to enhanced polyplex stability against salt induced stress [99]. Shorter peptides consisting of only six lysines mediated sufficient stability and notable gene transfer after cysteine dependent cross-linking [48]. With the help of convergent solid-phase synthesis, defined bioreducible polylysine derivatives comprising up to 74 lysines could be synthesized [100], revealing the possibilities of solid-phase synthesis.

In another approach, Rice and colleagues introduced acridine onto the ε-amine of a lysine suitable for SPS [101]. These acridinylated oligolysines complexed pDNA by charge dependent ionic interaction and also by polyintercalation (Figure 3B) [101–104].

Analogous to classical peptide synthesis, artificial building blocks such as triethylene tetramine or fatty acids were incorporated together with natural amino acids [53,66,67]. Wang et al. [66] designed a novel lipopeptide system (EHCO) based on (1-aminoethyl)iminobis [N-(oleoylcysteinylhistinyl-1-aminoethyl) propionamide] (Figure 3C) containing cysteines and oleic acids for siRNA nanoparticle stabilization, histidines for endosomal protonation, and (promoted by the fatty acids) endosomal membrane destabilization. The use of completely unnatural building blocks in SPS nucleic acid carriers was first introduced by Hartmann, Börner, and colleagues [68–74]. By alternating coupling of diamines (3,3′-diamino-N-methyl-dipropylamine or a bis-tBoc-protected spermine) and a diacid (succinic acid anhydride), the first sequence-defined oligo(amidoamines) were yielded. Optionally, disulfide linkage or a terminal PEG chain was introduced, and the sequence-defined oligomers were used for pDNA polyplex formation. Schaffert et al. [86] optimized the use of artificial amino acids for sequence-defined oligomer synthesis (Figure 3D). The design of the building blocks was based on the proton sponge diaminoethane motif of PEI. Triethylentetramine, tetraethylenpentamine, or pentaethylenhexamine were used with tBoc protection groups at the secondary amines and converted into artificial amino acids by introducing succinic acid onto one of the terminal primary amines, and Fmoc on the other primary amine [86,87]. With these novel artificial amino acids, oligomers were generated benefiting from the nucleic acid binding abilities as well as exhibiting a proton sponge effect, well known from PEI [23,105]. In combination with commercially available Fmoc α-amino acids, fatty acids, and also other artificial blocks introducing bioreducible breaking points [64], more than 1000 oligomers with different topologies for pDNA as well as siRNA delivery were synthesized. These topologies include linear [28,75], two-arm [75], three-arm [75,77,78,88], four-arm [75,87,106], comb architectures [29] as well as compounds with two cationic arms attached to a third arm of polyethylene glycol (PEG) of defined length and a targeting ligand (Figure 3E) [78,88,106–110].

With the precision of chemical design, in contrast with classical polymers like PEI or polylysine, oligomers could be generated to address simple questions on structure–activity relationships. For example, linear sequences of the building block Stp (succinyl tetraethylene pentamine, exhibiting three protonatable nitrogens per repetition) were prepared and the effect of increasing molecular weight of PEI-like oligomers on formed pDNA polyplexes could be investigated [28]. Very clearly, oligomers containing 20 Stp units (i.e. 100 nitrogen backbone) demonstrated good pDNA compaction, high marker gene transfer (6-fold higher than with gold standard LPEI 22kDa) in cell culture transfections, and an oligomer length-dependent 10-fold lower cytotoxicity than LPEI (containing in average an approximately 500 nitrogen backbone).

For further polyplex stabilization, terminal cysteines [75,77] or twin cysteines [111,112] served the formation of bioreducible disulfides. Optionally, further nanoparticle stabilization by incorporation of hydrophobic domains consisting of saturated as well as unsaturated fatty acids [64,75,77,113,114], or tyrosine trimers [64,113] at peripheral or central positions lead to T-shaped, i-shaped, or U-shaped oligomers with favorable properties for siRNA delivery *in vitro* as well as *in vivo*. Also the influence of different lengths of shielding agents in PEGylated two-arm structures on pDNA compaction and polyplex stability was examined [88]. An increased length of PEG (from 12 to 24 ethylene oxide units), resulting in a decreased polycation to PEG ratio, led to less compacted pDNA polyplexes as compared with unshielded polyplexes.

Remy and colleagues [115–117] took a completely different approach, designing a covalent incorporation of cationic carrier elements into nucleic acids. They adapted oligonucleotide SPS for synthesizing oligospermine–siRNA conjugates, which mediated efficient gene silencing in the absence of any other carrier. In course of their work, also lipidic elements were incorporated for improved efficacy [118].

## Polyplex shielding

**Figure 4:**
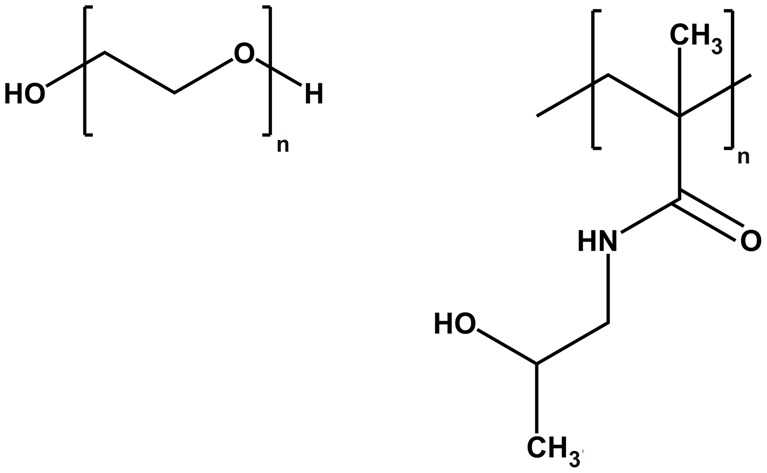
Chemical structures of the most prominent agents used for shielding Left: polyethylene glycol (PEG), right: poly(N-(2-hydroxypropyl)methacrylamide) (HPMA).

Nucleic acid complexation usually requires an excess of cationic charged carrier and thereby usually results in formation of nanoparticles with positive surface potential. This positive charge often displays an advantage for gene transfer efficacy *in vitro* due to unspecific binding to negatively charged cell surfaces [119,120] or by facilitating endosomal escape [121,122]. In the extracellular space, however, positively charged polyplexes depending on the applied cationic carrier may mediate undesired interactions with the complement system, blood cells, or other blood components [123–126]. Introduction of a hydrophilic surface shielding domain into artificial carriers has shown to reduce these interactions. PEG represents the most prominent and well-established shielding agent and has been successfully used for shielding of polyplexes in numerous instances, including SPS-designed nucleic acid carriers [12,103,123,127–131]. But also poly(N-(2-hydroxypropyl)methacrylamide) (pHPMA) [132,133], hydroxyethyl starch (HES) [134], polysarcosine [135], or repeats of Pro-Ala-Ser (PAS) [88] have been investigated as alternative hydrophilic shielding agents (Figure 4).

For example, Fmoc-PEG_*x*_-COOH was directly integrated into sequence-defined carriers during SPS (compare Figure 3D) [76,78,108,109,136]. Using folate or methotrexate (MTX) as folate receptor (FR) targeting ligands, small unimolecular siRNA nanoplexes were generated, which demonstrated FR-dependent *in vivo* gene silencing, and in case of MTX also therapeutic antitumor activity [76,136].

Although PEGylation may greatly improve pharmacokinetics and biodistribution to tumor target tissue, it may also negatively affect nucleic acid compaction (previous section) and intracellular performance [88,137,138]. The length of the PEG chain, and consequently the ratio of hydrophilic to cationic polymer within the polyplex, controls characteristics like nucleic acid compaction, polyplex size, and stability [88]. In a recent report by Kos et al. [78], systemic c-Met targeted gene transfer of pDNA polyplexes was successful, but only if combination polyplexes of a ligand-PEG carrier with a non-PEGylated compaction carrier were applied. Alternatively, to avoid difficulties with nucleic acid compaction, PEG was also introduced after pDNA [133,139] or siRNA [140–142] polyplex formation (“post-PEGylation”). For siRNA delivery, this approach led to increased tumor-specificity of RNA delivery *in vivo*, but only if tumor-specific ligands (EGFR binding peptide [140], transferrin protein [141], or folate [142], see next section) were applied.

Reduced intracellular efficacy is the second problem of the so-called “PEG-Dilemma”. As previously shown for other carriers, this problem can be overcome by introducing a pH-labile shield [130,143–145]. Removal of the shield at endosomal pH in the endolysosomal compartment was found to recover transfection activity *in vitro* and *in vivo*, also for pDNA polyplexes of sequence-defined oligomers [133].

## Ligands for cellular targeting

After formulation, carriers loaded with nucleic acid have to be able to reach target cells. Physical concentration via adsorption, electrostatic interactions, and ligand–receptor interaction are possibilities for successful intracellular entry of vehicles. Nanoparticles, comprise nucleic acid and cationic core exhibiting target specific ligands, may facilitate specific binding to receptors expressed on the surface of target cells. Afterward, carriers can be taken up by the cell via receptor-mediated endocytosis [14]. When polyplexes are positively charged, unspecific ionic interactions can still reduce the value of targeting ligands. Hence, targeting ligands are introduced in combination with shielding agents described above. As mentioned, targeting ligands plus shielding agents can be included directly during the SPS, conjugated after the synthesis, or introduced after polyplex formation. Many different targeting ligands such as antibodies and their fragments, glycoproteins, peptides, and small molecules that can bind to receptors overexpressed in cancer or other target cells, have been investigated [13,146–149]. Up to now, several different receptor-targeted carriers based on SPS already showed favorable characteristics for enhanced nucleic acid delivery.

The group of Rice [150] designed an asialoglycoprotein receptor (ASGP-R) targeted carrier with triantennary galactose-terminated oligosaccharide as a ligand, which combined with the endosomalytical reagent chloroquine, enhanced DNA delivery on the HepG2 cell line. The same group showed receptor specific uptake of pDNA/polyacridine glycopeptides (Figure 3B). They introduced high-mannose N-glycane as a targeting ligand attached to modified forms of polyacridine peptides [151,152].

The ligand RGD (arginine–glycine–aspartic acid) is one of the most commonly used peptides for nucleic acid nanoparticle targeting cell–surface integrins [78,93,94,108,153,154]. RGD–oligolysine peptide in combination with lysosomatropic chloroquine or lipidic helper molecules mediated targeted nucleic acid delivery [55,59,93–95]. Leng et al. [59] developed a library of effective vehicles for siRNA delivery, branched peptides composed of histidines, and lysines (HK) with optionally attached RGD ligand. A promising integrin targeted siRNA delivery system, which showed efficient gene silencing in U87 glioma cells, was introduced by Wang et al. [154]. This system was based on (1-aminoethyl)iminobis [N-(oleoylcysteinylhistinyl-1-aminoethyl) propionamide] (EHCO), see Figure 3C. RGD was attached to siRNA nanoparticles via a PEG spacer. Analogously, bombesin was applied as another receptor ligand, which binds specifically to the gastrin-releasing peptide receptor, neuromedin B receptor, and the orphan receptor bombesin receptor subtype 3 that are overexpressed in various cancers. Systemic administration of the targeted nanoparticles loaded with anti-HIF-1α siRNA showed significant tumor growth inhibition *in vivo* [154].

Martin et al. [108] demonstrated ligand-dependent pDNA delivery by designing cyclic RGD-PEG-Stp 2-arm oligoaminoamides (Figure 3E); the same strategy was successfully developed for the targeting peptide B6, which was initially assumed to enhance uptake via the transferrin receptor (TfR) but later on was discovered as an TfR independent tumor cell uptake facilitator [155,156]. These initial conjugates were devoid of endosomal buffering histidines, therefore the presence of the endosomolytic reagent chloroquine was necessary for high level transfection. Subsequent work demonstrated a greatly improved transfection activity of PEGylated 2-arm structures upon incorporation of alternating histidines into the Stp carrier backbone [106]. This kind of oligomer, containing the peptide ligand cMBP2 binding to hepatocyte growth factor receptor/c-Met, showed enhanced gene delivery efficacy and target-specificity *in vitro* in HUH7 hepatoma and DU145 prostate carcinoma. Upon intravenous application *in vivo* in a hepatocellular carcinoma xenograft mouse model, specific and ligand-dependent gene transfer was detected, but only if combination polyplexes of a ligand-PEG carrier with a non-PEGylated compaction carrier were applied. Using a plasmid encoding the theranostic gene sodium iodide symporter (NIS), radioiodide-mediated tumor detection, and antitumoral activity were demonstrated [78,157].

In order to achieve improved selectivity and transfection activity, a dual-targeting concept, which simultaneously targets two different overexpressed receptors in tumors, was also investigated. Cyclic RGD peptide, B6 peptide, and the epidermal growth factor receptor targeting peptide GE11 were evaluated. In the investigated DU145 prostate cancer cell culture, which expresses all involved receptors, the most successful pDNA delivery was obtained by the combination of GE11 and B6 ligands [158]. EGFR targeting via peptide GE11 was also used for siRNA lipopolyplexes, which were surface-PEGylated with maleimide–PEG–GE11. These formulations showed potential for EGFR-specific siRNA and miRNA-200c delivery [140].

Transferrin (Tf) as an iron transport protein is targeting the transferrin receptor (TfR) overexpressed in many different malignant cells. Therefore, it was applied as ligand in pLys/pDNA polyplexes [159,160]. Previously, a Tf–pLys system was used for the preparation of IL-2 gene modified cancer vaccines in the first polyplex *ex vivo* human clinical gene therapy trial [161]. Tf–PEI conjugates were also shown to enhance gene transfection efficiency up to 1000-fold in TfR overexpressing cell lines [128,162–164]. A Tf–PEG-coated cationic cyclodextrin carrier was very effective in siRNA delivery, which was the basis for the first TfR-targeted *in vivo* siRNA human clinical trial [165]. Zhang et al. [166] combined sequence-defined, histidinylated 4-arm oligomers with Tf–PEI conjugates for efficient TfR-targeted pDNA delivery. An alternative TfR-targeted system was introduced by Prades et al. [167] with applying the retroenantio approach to a peptide that targets TfR; this was found capable to overcome the blood–brain barrier. Based on T-shaped lipo-oligomers, TfR-targeted siRNA polyplexes were generated by post-introduction of INF7 and PEG–Tf or PEG–TfR antibody (TfRab) onto the polyplex surface. These carriers mediated effective target-dependent gene silencing and potent tumor cell killing *in vitro*, as well as a tumor-target specific biodistribution *in vivo*, but limited *in vivo* stability [141].

Folic acid (FA), the vitamin with high-binding affinity to the FA receptor in many tumor types [168], was also effectively incorporated into 2-arm and 4-arm oligomers [106,107,109,169,170] or lipo-oligomers [142] for pDNA or siRNA delivery. FA–PEG–Stp 2-arms can formulate single influenza peptide INF7 conjugated-siRNA into very small nanoplexes [107]. The INF7 peptide was strictly required for endosomal escape. The analogous siRNA nanoplexes using MTX as targeting and cytotoxic ligand were able to cure mice from KB tumors after intratumoral application [76]. Combination of FA targeted PEGylated 2-arm oligomer with untargeted, 3-arm oligomer by directed disulfide exchange reaction resulted in generation of larger ∼100 nm TCP polyplexes, which enabled FA specific gene silencing *in vivo* also upon intravenous administration [170]. Optimization of FA–PEG containing carriers was extended in a library approach, evaluating 2-arms versus 4-arms, different building blocks, presence/absence of buffering histidines or polyplex-stabilizing tyrosine trimers. A two-arm folate-targeted oligomer containing histidines and tyrosine trimers was recognized as the most promising FA-containing carrier for the delivery of both pDNA and siRNA [109]. Folate receptor targeting by PEGylating siRNA lipopolyplexes was developed by Müller et al. [142] Tetra-γ-glutamyl FA had to be used as targeting ligand; PEGylation with standard FA–PEG (but not FA-free PEG) resulted in nanoparticle aggregation.

For targeting brain tumors, the blood–brain barrier (BBB) or at least the blood–tumor barrier presents a significant bottleneck. A combinatorial approach for effective glioma-targeted siRNA delivery was introduced by An and colleagues [171]. For siRNA lipopolyplex formation, a T-shaped oligoaminoamide was combined with an angiopep 2 (LRP-targeting peptide) attached via PEG to a sequence-defined 2-arm oligomer (compare Figure 3E). After intravenous delivery, receptor-enhanced accumulation in a brain tumor and enhanced gene silencing of a target gene were observed. Similarly, another glioma targeting ligand, I_6_P_7_, an interleukin-6 receptor binding peptide derived from IL-6, was included into a similar sequence defined carrier construct for glioma-targeted delivery of pDNA [110]. In this case, a histidinylated carrier version was applied and combined with a histidinylated compaction carrier analogously as described above for c-Met targeting [78]. *In vitro* and *in vivo* results demonstrated transfer across BBB as well as therapeutic antitumoral effects against the brain tumor when pING4 gene transfer was performed [110].

## Endosomal escape

**Figure 5:**
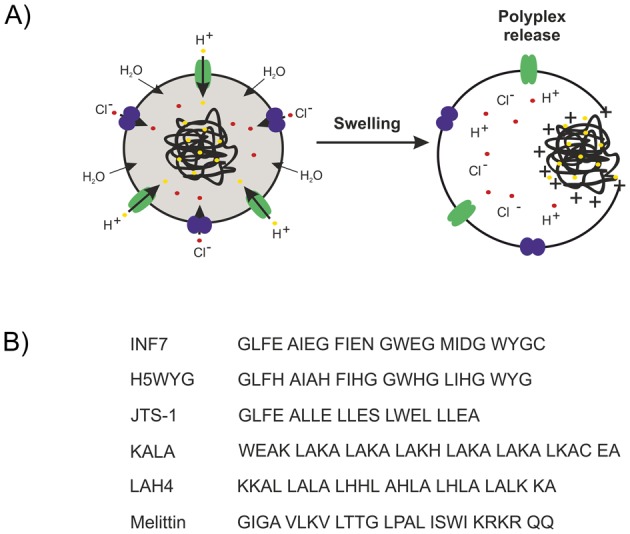
Strategies for endosomal escape (**A**) Schematic presentation of endosomal release by the proton sponge effect. Note that beyond osmotic swelling, direct destabilization of the phospholipid domain by the cationized polymer domains contributes to endosomal escape. (**B**) Membrane destabilization by amphiphilic lytic peptides.

Effective endosomal escape to release the entrapped polyplexes into the cytosol is an important event for successful nucleic acid delivery. Otherwise, nucleic acid will be digested during the conversion of endosomes toward lysosomes or recycled to the cell surface and removed out of the cell. Endosomes are intracellular vesicles and mostly serve for sorting, trafficking, and recycling of endocytosed material. Active transport of protons from the cytosol into the vesicle generated by the action of the proton pump ATPase is a reason for acidification of a series of vesicles. Based on the proton sponge hypothesis (Figure 5A), Jean-Paul Behr and colleagues [105] screened a series of “proton-sponge” polymers which exhibit weakly basic functionalities with p*K*_a_ values between physiological and endosomal pH. Thus during endocytic trafficking, such polymers would experience increase in protonation. Increased cationization and counterion concentration might be a reason for osmotic swelling and rupture of the endosomes membrane, causing the escape of polyplexes into the cytosol. Such considerations were the basis for the development of polyethylenimine (PEI) as transfection agent [23], or subsequent SPS-based oligoaminoamides [75,87] utilizing the aminoethylene motif of PEI. Uchida et al. [172] and later on Lächelt et al. [106] showed that oligoaminoethylene building blocks with even numbered amine groups (two or four protonatable nitrogens) have the highest buffer capacity around pH 5–6. Data accumulating during the last two decades rule out a purely osmotic effect for endosomal escape [121,126,143,173–176]. Direct interaction of protonated, cationized polymer domains with the endosomal phospholipid domain appear as essential for vesicle destabilization. In addition, free polycations (not bound to polyplexes) were found to critically contribute to gene delivery [177–180], and instead of complete lysis, only partial vesicle disruption was observed [176].

Nonprotonatable polymers such as polylysine can be converted into proton sponges. It is known that histidinylation of polylysine or PEI offers higher endosomal buffer capacity based on a p*K*_a_ around 6 of the imidazole groups; therefore, protonatable histidines were introduced into sequence-defined oligolysine-based carriers [48,57–61,78,106,109,181–185]. Consequently, total buffer capacities as well as nucleic acid transfer increased both *in vitro* and *in vivo*. Several groups reported about positive effect of histidines in the structures. Incorporation of histidines into a peptide of Cys-His-(Lys)_6_-His-Cys improved *in vitro* gene expression also in the absence of chloroquine as described by McKenzie et al. [48]. Read and colleagues reported efficient intracellular delivery of siRNA with histidine-rich reducible polycations [51]. The lab of Mixson developed a series of branched (HK) peptides containing lysines for nucleic acid binding and histidines for endosomal-buffering [57]. They further modified HK peptides of different length by adding histidine-rich tails. Thus, increased buffer capacity further improved transfection efficiency [60].

The proton sponge effect is not the only solution to overcome the endolysosomal entrapment. In fact, previous studies with (nonproton sponge) polylysine carriers already had shown that integration of fusogenic peptides (Figure 5B) such as influenza-derived INF1-7, JTS-1, or H5WYG into polylysine/pDNA polyplexes improved gene transfer significantly. The latter mentioned peptides mimic the functions of viral proteins and enable permeabilization of the endosomal membrane triggered by acidification of endosomes [186–188]. As reported by Dohmen et al. [107], the endosomolytic peptide INF7, originally designed as the glutamic acid-enriched analog of the influenza hemagglutinin membrane protein HA2 N-terminus, was coupled to the 5′-end of the siRNA sense strand, which maintains its silencing efficiency with increased endosomal escape when formulated into nanoplexes INF7 also greatly improved TfR-targeted siRNA lipoplexes when incorporated by post-modification of lipoplex surface [141] Artificial amphipathic cationic peptides such as KALA and LAH4, or derivatives of the bee venom melittin facilitated significantly improved gene transfer [189–191]. The latter peptides own two important properties for efficient gene transfer—possibility of DNA binding and destabilization of membranes. The positive charge of KALA allows electrostatic interactions with the negatively charged pDNA. However, the positive charged amphiphile KALA can also interact with the endosomal membrane and consequently can cause membrane leakage [189 ]. Next, partially mimicking the proton sponge activity of PEI and presence of histidine residues are responsible for improved endosomal escape in the case of LAH4 [190]. Boeckle et al. [191] showed that melittin–PEI conjugates can enhance gene transfer, but also cause high toxicity due to lysis of the plasma membrane. Therefore, modifications with acidic residues (glutamic acid or histidine) should allow high lytic activity at acidic pH to induce membrane destabilization in endosomes. Polyacridine peptides modified with melittin (by either a maleimide-Cys or a thiopyridine-Cys linkage) were used in pDNA transfection with efficacies as high as for PEI [101]. Also others peptides called cell-penetrating peptides (CPPs) promote endosomal escape, for example, PepFect6 [53] and PepFect14 [54].

In case of cationic lipoplexes, endosomal escape may occur through local, transient perturbations of the endosomal membrane by lipid mixing; cationic lipids possess the ability to form nonbilayer structures and charge neutral ion pairs with the negatively charged phospholipids (shift to the inner part of endosome caused by lipoplexes) [192]. Analogously, incorporation of fatty acids into polycation structures presents another option for generating amphiphilic characteristics that facilitate endosomal escape. The group of Lu generated lipo-oligomer carriers for pDNA and siRNA delivery, with two oleic acid residues triggering a pH-dependent disruption of lipid membranes [66]. Also Schaffert, Fröhlich, and colleagues generated lipo-oligomer carriers based on oligoaminoamides, which were modified with pairs of fatty acids incorporated at terminal lysine amines in i-, T-, or U-shaped topologies [75,77,114]. The type of incorporated fatty acid had more influence on the performance than the topology. Oligomers modified with the unsaturated (C18) fatty acids oleic acid and linoleic acids demonstrated best transfection efficiency due to endosomal pH-specific lytic activity. Furthermore, myristic acids (C14) caused high, but pH-independent lytic activity but also cytotoxic effects. Recently, Klein et al. [64] designed T-shaped oligomers containing a bioreducible disulfide bond between the cationic and lipid building block. Thus, the carriers would dissociate via GSH-mediated cleavage in the cytosol into nontoxic fragments leading to enhanced intracellular nucleic acid release while improving polyplex stability in the extracellular space. Using this strategy, bis-myristyl and bis-cholanic acid based lipo-oligomers should enable high lytic activity, high siRNA, delivery and silencing activity in the absence of cytotoxicity.

## Cargo release and nuclear delivery

Polyplex stability is a critical issue for extracellular delivery, where high stability is of highest importance; it also is a critical parameter in intracellular delivery and subsequent cargo release at the target site, where nucleic acid release or at least exposure in bioactive form is important. To mediate gene silencing, siRNA and miRNA need only to reach the cytoplasm for incorporation into the RISC complex. For pDNA, further transport through the cytoplasm toward the nucleus (before or after endosomal escape, with or without complexation with cationic carrier), entry across the nuclear envelope, and accessibility for transcription are required.

Events following endosomal escape (fate of the polymer, nucleic acid, and different sortings of endosome) are still poorly understood. In fact, cargo release and productive delivery very much depend on the specific cargo size, the carrier, cell type, and different intracellular routes [193,194]; it is impossible to provide a general statement on the fates. First of all, even with effective nanoparticle systems, endosomal release is a rare event and bottleneck in the delivery process, therefore subsequent steps are difficult to track [176,195,196]. Even with potent siRNA LNPs, only 1–3% of internalized siRNA molecules were delivered into the cytosol [197,198]. For these LNPs, a narrow window of siRNA release from maturating endosomes approximately 5–15 min after internalization was observed. Releasing endosomes were recognized by cytosolic galectin-8/-9, which target them for autophagy [198]. Moreover, exocytosis of recycling siRNA nanoparticle-loaded vesicles was identified as a limitation [199]. In a different study, gene silencing potency correlated with intracellular siRNA lipopolyplex stabilization instead of early endosomal exit [200].

Only few studies have been performed comparing lipoplexes (e.g. lipofectamine) and polyplexes (with PEI), but significant differences were observed in the intracellular delivery steps [176,192,201,202]. Endosomal escape of lipoplexes by mixing of cationic lipids with the negatively charged phospholipids of endosomal membranes should release nucleic acids in lipid-free form [192,203]. For some lipoplex-mediated transfection using oligocationic lipids, however, despite effective nuclear delivery of pDNA, an insufficient release and availability for transcription were reported as possible limitation for gene transfer [204]. For polyplexes, the site of release from polycations such as PEI is even less clear, although delivery of small polyplexes was been reported. Interestingly, free PEI was found to not only enhance endosomal escape, but also assist in transfer of pDNA into the nucleus (by ∼5-fold), enhance the pDNA-to-mRNA transcription efficiency (by ∼4-fold), and facilitate the nucleus-to-cytosol translocation of mRNA (by 7–8-fold) [180].

Nuclear import is a crucial size-dependent process, and presents the next important barrier for delivery of larger nucleic acids such as pDNA [12]. The nuclear pore complex (NPC) only allows the passage of small molecules such as oligonucleotides [176,205,206]. whereas polyplexes greater than ∼50 nm do not have this capacity. In that case, nuclear entry relies on nuclear membrane breakdown during cell division process [207]. The importance of the nuclear import step has been demonstrated in cell cycle studies. Transfection efficiency of branched PEI polyplexes was strongly enhanced in the G2/M phase, when the nuclear envelope breaks down. In contrast, linear PEI polyplexes showed lower cell cycle dependence. The same was observed for c-Met-targeted, PEG shielded minicircle (MC) DNA polyplexes, which were well compacted into 35–40 nm rod structures by tyrosine trimer-containing Stp oligoaminoamides [208]. Conjugation of short cationic nuclear localization signals (NLS) peptide for an active, targeted transport through the NPC has been evaluated as a possible solution for cell-cycle independent gene transfer [12,18–20,209]. The exact conditions to successfully utilize the properties of NLS peptides are still unclear and therefore only a small number of carriers which could reach the nucleus have been described [153,210–215]. Further optimizations of nuclear import are required for improved pDNA delivery into nondividing cells.

## Challenges of *in vivo* delivery

*In vivo* delivery faces several additional hurdles. As mentioned in previous sections, polyplex shielding and receptor-targeting are possible measures to avoid undesired reactions such as innate immune responses and to provide some specificity upon systemic administration, for example, in passive or active tumor targeting [123,124]. For this purpose, numerous targeting ligands for various cell surface receptors have been evaluated *in vivo* [9,55,62,65,78,110,128,156,157,165,171,200,216–221]. The polyplex size may be at least as crucial for *in vivo* performance as the ligand selection; for example, free siRNA or nanoparticles exhibiting a size of approximately 6 nm are quickly cleared by the kidney [107,222]. Passive targeting of blood-circulating nanoparticles by the EPR effect (enhanced permeability and retention of tumor tissue) offers polyplexes of a size of 20 nm up to 400 nm distribution into solid tumors via leaky vasculature [223,224]; the EPR effect, however, can be tumor type- and patient-specific and also heterogeneous within tumors. Polyplex delivery may be ineffective in less vascularized tumors [225]. For tumors such as stroma-rich pancreatic cancer, only smaller nanoparticles were effective [226]. Despite the many efforts, the efficiency of tumor targeting is still low; Chan and colleagues reviewed published work and concluded that on average only 0.7% of the dose is accumulating at the target tumor site [227].

Apart from targeting, shielding, and nanoparticle size, the stability of polyplexes is an additional challenge for *in vivo* performance; thus, additional measures such as bioreversible internal covalent cross-linkage of polyplexes or incorporation of bioresponsive domains into carriers for noncovalent stabilization have to be investigated [125,139,228–231]. Another critical aspect for *in vivo* gene delivery is the reduction of polyplex- and carrier-triggered toxicity. The transfection efficiency of frequently used high molecular weight PEI goes hand in hand with an N/P dependent cytotoxicity; mechanistic details are reviewed in Hall et al. [126]. Nevertheless, linear PEI has already been developed for clinical application with encouraging results [232]. The therapeutic window in systemic administration and wider therapeutic use still would strongly benefit from a reduced carrier cytotoxicity. In that view, degradable PEI analogs are highly desirable [233]. In this regard, SPS offers excellent opportunities to design structurally precise carriers with cysteine residues for cleavable linkages. During polyplex formation, the cysteines form bioreducible disulfides and thus enhance stability in the extracellular part of the gene delivery process. When having reached the bioreductive environment of the cytosol, bioreducibility of the polyplexes enhances cargo release and also cause fragmentation of the carriers into smaller less toxic pieces [46,48,50,51,75,100,111].

## Conclusion and prospects

Over the past decades, significant progress has been made in the field of nucleic acid delivery vehicles. Sequence-defined macromolecular carriers synthesized by SPS play a significant role in this development. As several different extracellular and intracellular barriers must be overcome for successful transfer, the multifunctional nature of such carriers is of greatest importance. Carriers need to be dynamic [234,235]. On the one hand, stability of complexes is important at the time of extracellular delivery steps, while on the other hand, the carrier must release therapeutic nucleic acid after delivery inside the cell. SPS offers excellent opportunities to develop structural precise carriers, which is crucial for establishing appropriate structure–activity relationships. Still further optimization of delivery carriers is required. A better understanding of structures characteristics in nucleic acid complexation, target cell recognition, endosomal escape, nuclear delivery, and transgene expression or toxicity is necessary. In sum, sequence-defined delivery carriers containing natural and/or artificial building blocks represent a valuable part in the development of “smart” delivery systems, which will have great impact in the medicine of tomorrow.

## Abbreviations

DCC: dicyclohexylcarbodiimide
DCM: dichloromethane
GSH: Glutathione
HBTU: 2-(1H-Benzotriazol-1-yl)-1,1,3,3-tetramethyluronium hexafluorophosphate
HOBt: Hydroxybenzotriazole
HF: hydrofluoric acid
INF7: glutamic acid-enriched analogue of the influenza hemagglutinin membrane protein HA2
LPEI: linear polyethylenimine
PEG: polyethylene glycol
PEI: polyethylenimine
PyBOP: Benzotriazole-1-yl-oxy-tris-pyrrolidino-phosphonium hexafluorophosphate
SPS: solid-phase assisted synthesis
TFA: trifluoro acetic acid
RISC: RNA-induced silencing complex
